# Cognitive impairments associated with medial temporal atrophy and white matter hyperintensities: an MRI study in memory clinic patients

**DOI:** 10.3389/fnagi.2014.00098

**Published:** 2014-05-27

**Authors:** Eduard J. Overdorp, Roy P. C. Kessels, Jurgen A. Claassen, Joukje M. Oosterman

**Affiliations:** ^1^Department of Psychiatry, Gelre Medical CentreZutphen, Netherlands; ^2^Donders Institute for Brain, Cognition and Behaviour, Radboud University NijmegenNijmegen, Netherlands; ^3^Department of Medical Psychology, Radboud University Medical Center–Radboud Alzheimer CenterNijmegen, Netherlands; ^4^Department of Geriatric Medicine, Radboud University Medical Center–Radboud Alzheimer CenterNijmegen, Netherlands

**Keywords:** gray matter, global atrophy, global cognition, executive function, episodic memory

## Abstract

In this retrospective study, we investigated the independent effects of white matter hyperintensities (WMH) and hippocampal atrophy on cognitive functions in a broad sample of patients seen in a memory clinic. To ensure generalizability, these associations were examined irrespective of diagnosis and with minimal exclusion criteria. Next to these independent effects, interactions between WMH and hippocampal atrophy were examined. Between January 2006 and September 2011 a total of 500 patients visited the memory clinic, 397 of whom were included. Magnetic resonance images of 397 patients were visually analyzed for WMH, medial temporal atrophy (MTA), and global atrophy. We evaluated the association of WMH and MTA with the following cognitive domains: global cognition, episodic memory, working memory, executive function and psychomotor speed. Main effects and interaction effects were examined by means of correlation and regression analyses. In the regression analyses, we controlled for potential confounding effects of global atrophy. The correlational results revealed that WMH were associated with global cognition, executive function and psychomotor speed, whereas a trend was found for episodic memory. MTA was associated with all these four cognitive domains; an additional trend was observed for working memory. Hierarchical regression analyses revealed main independent effects of MTA for episodic memory, executive function, psychomotor speed and global cognition; WMH were only associated with global cognition. The interaction between MTA and WMH was significant for episodic memory only. This study demonstrates that predominantly MTA is an independent predictor not only for memory function, with which is it classically associated, but also for global cognition and executive function. Taken together, MTA may be an important correlate of cognitive deficits found in people attending the memory clinic.

## INTRODUCTION

Medial temporal atrophy (MTA), and in particular hippocampal atrophy, is one of the earliest changes seen in the brains of patients with Alzheimer’s Disease (AD), and its presence has proven to be a sensitive marker for the diagnosis of AD (Braak and Braak, 1991). MTA is also an early marker of AD on magnetic resonance imaging (MRI). Longitudinal studies found that MTA was already present in preclinical AD and in this preclinical stage the rate of hippocampal volume loss also predicted the progression to AD dementia (Twamley et al., 2006). Studies of the relationship between morphological and cognitive measures of AD have demonstrated that MTA, especially of the hippocampus, correlates with episodic memory impairment in AD (de Leon et al., 1997). Other cognitive abilities are affected as the neuropathological changes of AD spread from limbic structures to neocortical association areas (Braak and Braak, 1998). White matter hyperintensities (WMH) are also commonly observed in patients with AD, although their clinical relevance is not well understood (Barber et al., 1999). Some studies have reported that AD patients with WMH have slower cognitive processing speed and greater executive dysfunction than AD patients without WMH (Amar et al., 1996; Libon et al., 1998). Other studies found no substantial neuropsychological differences between patients with or without WMH (DeCarli et al., 1996; Doody et al., 1998; Bartrés-Faz et al., 2001). Compared to the contradictory results regarding the correlations between WMH and cognitive dysfunction, studies examining MTA have shown a more consistent association with cognitive deficits, particularly with memory. For example, MTA correlates with impairments of verbal memory in elderly with cognitive dysfunction (Kohler, 1994; Laakso et al., 1995). Even though there are many studies on cognitive impairments related to either MTA or WMH, only a few studies have assessed the independent effects of MTA and WMH concurrently on cognitive functioning. In one study on AD patients, gray matter volume and WMH were independently related to overall cognitive decline (Stout et al., 1996). A recent study examined the independent effects of WMH and MTA on various cognitive functions in AD patients (Shim et al., 2011), and found that the cognitive deficit profiles related to WMH were rather dissimilar from those associated with MTA. WMH were more related to attention and executive functioning, while MTA was more associated with visuospatial function, language, and memory. Another important finding was that interactions exist between WMH and MTA with regard to the effects on cognitive functions, suggesting that they synergistically contribute to cognitive impairment in AD.

Although these studies point toward associations of both WMH and MTA with cognitive function, several factors limit interpretation of these results. Most studies examined the effects of WMH and MTA in isolation, not taking into account the strong association that exists between these MRI correlates. Furthermore, the majority of these studies were performed in strictly defined groups of patients, e.g., only AD patients, in tertiary referral centers, which likely leads to referral bias. Additionally, these studies used extensive exclusion criteria, thereby risking selection bias. It is therefore currently unknown whether these findings can be generalized to a broad sample of patients seen in general memory clinics. Finally, most of these studies used global measures of cognitive dysfunction, lacking a detailed investigation of memory or executive function with well-defined test batteries.

Thus, the first goal of this study was to investigate if there is an independent effect of WMH and MTA on various cognitive deficits in a broad sample of patients seen in a memory clinic, regardless of diagnosis and without the use of extensive exclusion criteria. The second goal of this study was to investigate if there is an interaction effect of WMH and MTA on various cognitive deficits in this patient sample.

## MATERIALS AND METHODS

### PATIENTS

**FIGURE 1:**
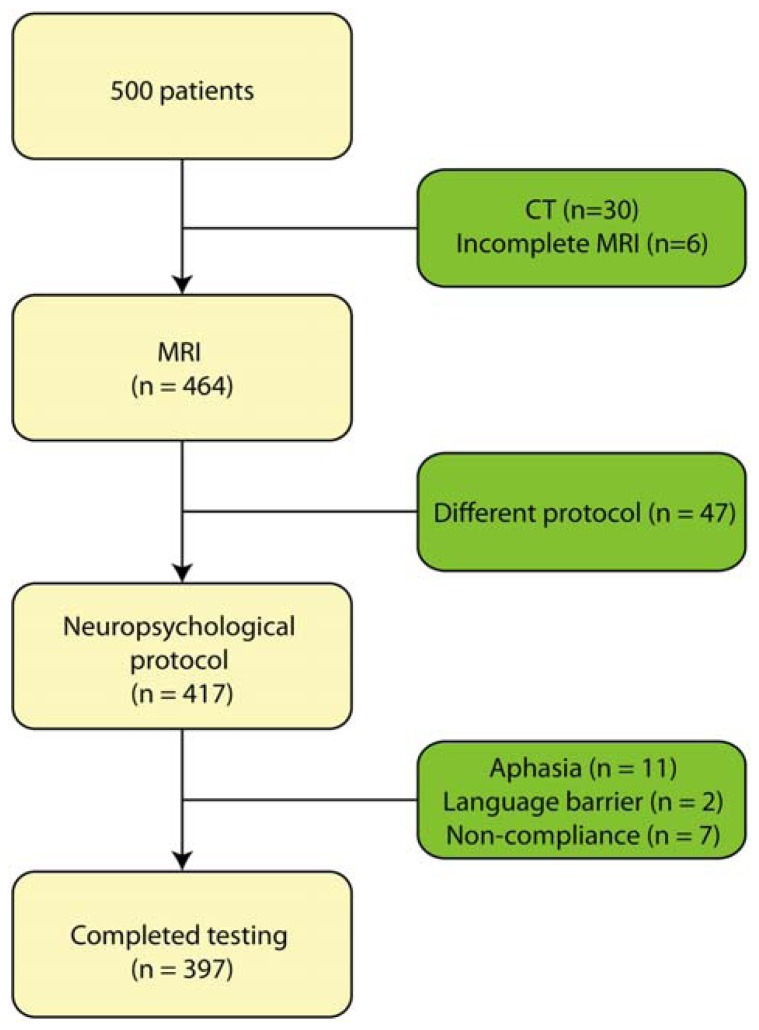
**Flowchart of the included patients**.

This retrospective study focused on patients who presented with memory complaints to the memory clinic at Zutphen Medical Center, Zutphen, The Netherlands, a general hospital. The majority of the patients were referred by the general practitioner. All had memory problems confirmed by a caregiver. The study population consisted of 500 consecutive patients who had been referred to the Zutphen memory clinic between November 2004 and September 2011. All patients underwent an elaborate work-up consisting of a general physical and neurological examination, blood screening, structural neuroimaging consisting of magnetic resonance imaging (MRI), and electroencephalogram as well as an extensive neuropsychological examination. Patients were excluded if they presented with a hearing impairment or visual impairment, or were too aphasic to complete the neuropsychological assessment. Patients who underwent CT-scan instead of MRI, because of claustrophobia or metal particles, were excluded from the sample (*n* = 30); MRI-data was furthermore not complete for six patients. In addition, neuropsychological test results were not available (due to communication difficulties resulting from Dutch not being the patient’s mother tongue, *n* = 2; severe aphasia, *n* = 11; or non-compliance, *n* = 7) or the test protocol differed systematically because of a younger age (*n* = 47) (see **Figure 1**). Hence, data of 397 patients were used for the analyses. The following variables were recorded: age at study enrolment, sex, level of education, vascular risk factors including hypertension, diabetes mellitus, cardiac disease and a history of stroke. Diagnoses were made in multidisciplinary consensus meeting according to the DSM-IV. At present, cerebrospinal fluid (CSF) assessment is not part of the routine diagnostic work-up for dementia in the Netherlands. However, in case no consensus was reached regarding the diagnosis, CSF was collected from the patient and taken into account in the diagnostic process. The following diagnoses were established: dementia due to Alzheimer’s disease (*n* = 98), mild cognitive impairment (*n* = 75), vascular dementia (*n* = 27), frontotemporal dementia (*n* = 11), Lewy body dementia (*n* = 6), Parkinsonism disorders (*n* = 16), stroke (*n* = 8), vascular cognitive impairment (*n* = 5), neurological disorder (e.g., encephalitis; *n* = 11), dementia (*n* = 2), other neurodegenerative disorder (e.g., progressive aphasia; *n* = 6), Korsakoff’s syndrome (*n* = 3), or a psychiatric disorder (e.g., depression; *n* = 29). For 40 patients, evidence of cognitive impairment was established, but no diagnosis could yet be made. One patient revealed mental retardation and one was diagnosed with post-operative cognitive impairment. Finally, 58 people did not show any sign of cognitive impairment and, hence, received no diagnosis. The level of education was classified using the system of Verhage (1964), ranging from 1 (less then primary school) to 7 (university degree). Characteristics of these patients are presented in **Table 1**.

**Table 1:** Characteristics of the patients.

	Patients
Age	75.82 (8.06)
Education	4.00 (1–7)
Sex (M/F)	175/222
WMH	1.50 (0–3)
MTA	1.00 (0–4)
GA	1.00 (0–3)

*Mean (±SD) is presented for age together with median (range) for education, WMH, MTA, and GA. Frequencies are presented for sex distribution.*

### NEUROPSYCHOLOGICAL ASSESSMENT

The neuropsychological test battery was designed to screen the majority of cognitive functions, including measures of orientation, memory, attention, language, executive functioning, and psychomotor speed. Two experienced neuropsychologists completed all neuropsychological examinations, and were blinded to all medical information at time of assessment. All tests were administered during a single session, with a fixed order of administration designed to minimize the introduction of any new verbal stimuli during delay periods for verbal memory tests. Testing was done on the same day as MRI scanning as part of the routine screening process. Tests were employed tapping the following domains: global cognition, working memory, episodic memory, executive function, and psychomotor speed. Multiple tests were used to assess each domain, with the exception of psychomotor speed, which was measured with a single test. 

Global cognition was assessed with the cognitive screening test (CST) and the mini mental state examination (MMSE: Folstein et al., 1975). The CST is a twenty-item orientation questionnaire that is commonly used to evaluate cognitive impairment (De Graaf and Deelman, 1991). Severity of cognitive impairment was assessed using the MMSE, a short screening instrument for cognitive functioning.

For working memory, the digit span forward and backward tests were used (WAIS-III, digit span, Wechsler, 1987), which require patients to repeat series of numbers increasing in length in the same or reversed order.

For episodic memory, the visual association test (VAT; Lindeboom et al., 2002), the 8-word test and a prose recall test were used. The VAT material consists of six association cards showing two interacting objects that are not commonly connected in daily life (for example an ape holding an umbrella), and six cue cards showing only one of the objects (e.g., the ape). At first, patients are shown the six cue cards with one of the objects, followed by the association cards with the previously seen object plus an interacting object. Patients are asked to name the object on the cue cards, and subsequently to name each pair of interacting objects on the association cards. Recall is tested immediately following presentation, by showing the cue cards again and asking what object is missing. One point is awarded if the response is sufficiently clear to distinguish the target object from the other objects used in the test; the series is then repeated (total score 0–12). In the prose recall test (Lezak et al., 2004), a short 20-item story is read to the patient, who is instructed to repeat as many items as possible immediately after presentation and after an interval of 10–15 min. Episodic Memory was furthermore assessed with the eight-word test of the Amsterdam dementia-screening test (de Jonghe et al., 1994), which includes a list of eight words to be recalled immediately after each of the five verbal presentations, as well as after a 10-min delay by means of a free recall and a recognition test.

Executive functioning was measured by using the trail making test part A and B (TMT; Reitan, 1958), the frontal assessment battery (FAB; Dubois et al., 2000), and the Meander task of the Amsterdam dementia-screening test. The TMT-A consists of 25 encircled numbers positioned randomly on a sheet of paper. The patient is required to subsequently connect these numbers. The more complex part B requires patients to alternate between letters and numbers (e.g., 1-A-2-B-3-C-…) and was used to evaluate cognitive flexibility. For both conditions, the time required for completion is recorded; the TMT-B ratio score (TMT-B/TMT-A) was used for the analyses. The FAB is a short cognitive and behavioral test battery consisting of six subtest: (1) similarities (conceptualization); (2) lexical fluency (mental flexibility); (3) motor series (programming); (4) conflicting instructions (sensitivity to interference); (5) go/no go (inhibition control); and (6) prehension behavior (environmental autonomy). Each subtest was rated from 0 to 3. The total score ranges from 0 to 18 (Takagi et al., 2002). The Meander, a figure of two alternating elements which the subject has to continue, is sensitive to perseverations characteristic for frontal disorders. Finally, the TMT-A completion time was included as a measure of psychomotor speed.

Ongoing efforts to improve the neuropsychological screening procedure resulted in a slightly varying content of the neuropsychological battery over the years. The following neuropsychological tests were applied since the establishment of the memory clinic in November 2004: TMTA+B; the eight-word test; the CST; the MMSE; the Prose recall test; the Digit Span Forward and Backward, and the Meander. In subsequent years the VAT (June 2005) and FAB (August 2007) were added to the neuropsychological battery. In addition, some patients could not complete all tests. As a result of both factors the available data varied per test: 253 patients completed the TMT-A, 169 patients completed the TMT-B, 371 the eight-word test, 397 the CST, 396 the MMSE, 326 the Digit Span Forward and Backward, 391 the Meander, 350 the Prose recall test, 378 the VAT, and 338 the FAB.

### MRI PROTOCOL

Brain MRIs were obtained with a 1.5 Tesla GE-Signa Horizon LX scanner. The same scanner was used for all patients. A standardized imaging protocol consisting of whole brain axial and coronal fluid-attenuated inversion recovery (FLAIR; TR 10,000 ms, TE 160 ms), sagittal T1 (repetition time TR 300 ms, TE 4 ms) and axial T2-weighted (TR 6500 ms, TE 105 ms) images was used. The slice thickness for all images was 5 mm with a 2 mm gap.****

The protocol of MRI acquisition for this study included the T1-weigthed axial, the T2-weigthed axial, the fluid-attenuated inversion recovery (FLAIR), and the T1-weigthed coronal images. The assessment of WMH, MTA, and global atrophy was performed by two experienced independent observers, blinded to the clinical diagnoses.

On the T2-weigthed axial and FLAIR images, we evaluated the severity of WMH according to the modified criteria of Fazekas, using a 0–3 rating scale (Fazekas et al., 1987). T1-weigthed coronal images were employed to rate MTA, using a 5-point rating scale (0–4) which is based on the width of the coronal fissure and the temporal horn and the height of the hippocampal formation (Scheltens et al., 1992). Global atrophy score is the mean score for cortical atrophy throughout the complete cerebrum, and was rated on a 4-point rating scale (0–3; Scheltens et al., 1997). These scores were based on the sulcal width and gyral thinning in the frontal, temporal and parieto-occipital lobes. For rating global atrophy the T1-weigthed axial, the T2-weigthed axial, FLAIR, and the T1-weigthed coronal images were used.

### STATISTICAL ANALYSES

Statistical analyses were performed using the SPSS software package version 19. For all analyses, the statistical significance was set at *p* < 0.05. To reduce the number of statistical analyses, cognitive domain scores were calculated. For this, the separate neuropsychological test scores were *z*-standardized. Next, average scores were calculated resulting in the following cognitive domains: global cognition (based on the MMSE and the CST-20), episodic memory (based on the VAT, 8-word test–immediate and delayed recall, prose immediate and delayed recall), working memory (based on digit span forward and backward) and executive function (based on TMT-B ratio score, Meander, FAB). Psychomotor speed was based on the individual TMT-A score. In case of missing neuropsychological test scores, the available test scores were used to calculate the specific domain.

First, Spearman rank correlations were calculated to explore the main associations between the cognitive functions, WMH and MTA. Next, to determine the unique contributions of WMH and MTA and the interaction between these two neuroimaging variables, the following steps were taken. WMH were dichotomized into no or mild WMH (score <2) and moderate to severe WMH (score ≥2); a similar approach was used to dichotomize MTA (no or mild MTA, score <2; moderate to severe MTA, score ≥2). Hierarchical multiple linear regression analyses were performed to examine the contribution of WMH and MTA to neuropsychological test performance. Global atrophy, dichotomized into no or mild atrophy (score <2) and moderate to severe atrophy (score ≥2), was entered first, to ascertain that effects of MTA and WMH reflect more than simply underlying global atrophy. Next, main effects of WMH and MTA were entered. Finally, the interaction between WMH and MTA was entered to explore whether the combination of severe WMH with severe MTA was related to worse neuropsychological test performance.

In addition to the interaction between WMH and MTA, similar hierarchical multiple linear regression analyses were performed for the interaction between WMH and GA and between MTA and GA. These analyses were performed separately for each interaction term in order to reduce multicollinearity among the predictors.

## RESULTS

No or mild WMH and MTA was present in 120 patients, moderate/severe MTA combined with no/mild WMH was present in 58 patients, moderate/severe WMH with no/mild MTA was present in 78 patients, whereas 141 patients had combined moderate/severe MTA and WMH.****

### CORRELATION BETWEEN COGNITIVE PERFORMANCE, WMH AND MTA

The result of the correlational analysis is presented in **Table 2**. WMH were associated with global cognition, executive function, and psychomotor speed, but there was only a trend (*p *= 0.07) for episodic memory. MTA was associated with these four cognitive domains; an additional trend was observed between MTA and working memory (*p *= 0.07).

**Table 2:** Correlations between the cognitive domain scores, WMH and MTA.

	Global cognition	Episodic memory	Working memory	Executive function	Psychomotor speed
WMH	-0.176^**^	-0.091	-0.017	-0.115^*^	-0.232^**^
MTA	-0.237^**^	-0.202^**^	-0.101	-0.212^**^	-0.287^**^

Spearman rank correlations were calculated. A higher cognitive domain score represents better performance. MTA: medial temporal lobe atrophy; WMH: white matter hyperintensities. ^*^p < 0.05, ^**^p < 0.01.

### MAIN AND INTERACTION EFFECTS OF WMH AND MTA

The cognitive domain scores were not normally distributed; we therefore examined which type of transformation (square root, logarithmic, or rank-based inverse normal transformation) resulted in the optimal skewness and kurtosis values; the goal here was to use the minimum amount of transformation (Osborne, 2002). Consequently, square root transformation was used for the working memory domain, natural logarithmic for the global cognition domain and the psychomotor speed score, and inverse normalization for the executive function and memory domains. Hierarchical regression analyses (see **Table 3**) revealed significant main effects of MTA for memory, executive function, psychomotor speed, and global cognition; WMH were only associated with global cognition. The interaction between MTA and WMH was significant for memory only.

**Table 3:** Main and interaction effects of WMH, GA, and MTA in relation to the different cognitive domain scores.

	Global cognition	Episodic memory	Working memory	Executive function	Psychomotor speed
**Step 1**
GA (β)	-0.140^**^	-0.108^*^	-0.062	-0.187^***^	-0.199^**^
**Step 2**
GA (β)	-0.077	-0.060	-0.046	-0.154^**^	-0.096
MTA (β)	-0.140^**^	-0.153^**^	-0.050	-0.116^*^	-0.238^***^
WMH (β)	-0.081	-0.007	-0.001	0.012	-0.072
**Step 3**
GA(β)	-0.080	-0.064	-0.045	-0.157^**^	-0.096
MTA (β)	-0.242^***^	-0.292^***^	-0.150	-0.219^**^	-0.202^*^
WMH (β)	-0.165^*^	-0.121	-0.086	-0.073	-0.045
MTAxWMH (β)	0.175	0.238^*^	0.173	0.177	-0.060
**Step 3**
GA(β)	-0.090	-0.268^**^	-0.165	-0.348^***^	-0.059
MTA (β)	-0.140^**^	-0.148^**^	-0.043	-0.112^*^	-0.240^**^
WMH (β)	-0.086	-0.086	-0.047	-0.062	-0.059
GAxWMH (β)	0.017	0.275^**^	0.157	0.256^*^	-0.048
**Step 3**
GA(β)	-0.171	-0.243^**^	-0.164	-0.255^**^	-0.010
MTA (β)	-0.179^**^	-0.229^***^	-0.096	-0.158^*^	-0.206^**^
WMH (β)	-0.078	-0.002	0.005	0.014	-0.077
MTAxGA (β)	0.128	0.251^*^	0.158	0.137	-0.115

Results of the hierarchical regression analyses with forced entry. For each cognitive domain, three analyses were performed, one for each interaction term. A higher cognitive score represents better task performance. GA: global atrophy; MTA: medial temporal atrophy; WMH: white matter hyperintensities. ^*^p < 0.05, ^**^p < 0.01, ^***^p < 0.001.

**FIGURE 2:**
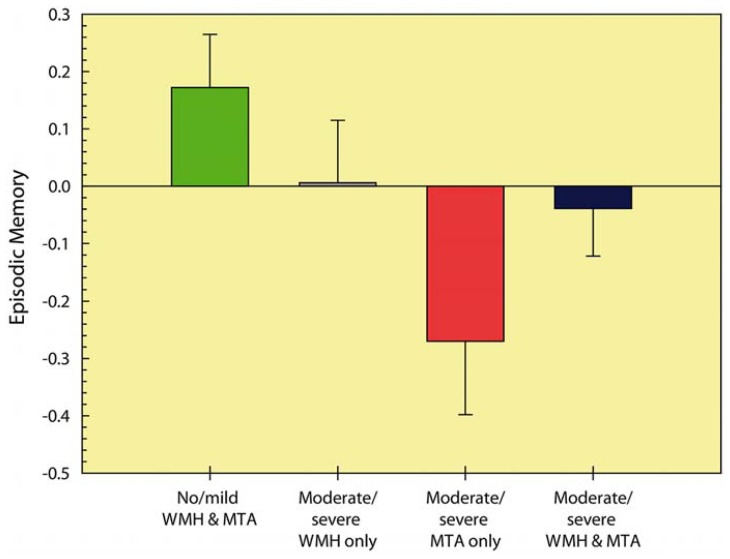
**Episodic memory performance of the different MTA and WMH severity groups.** Higher cognitive scores represent better performance. MTA: medial temporal atrophy; WMH: white matter hyperintensities.

Further inspection of this interaction effect while controlling for age (see **Figure 2**) revealed that memory performance was lowest in the group with severe MTA, followed by the group with combined severe MTA and WMH. *Post hoc* comparisons (with Bonferroni correction) revealed that compared to group with no or mild WMH and MTA, performance was worse in the moderate/severe MTA group (*p *< 0.001) and in the group with concurrent moderate/severe MTA and WMH (*p *< 0.05). No other between-group comparisons were significant.

Hierarchical regression analyses on the interaction effect between WMH and GA (see **Table 3**) revealed significant effects of this interaction term for episodic memory and executive function.

Further inspection of these interactions, while controlling for age, revealed that memory performance was lowest in the group with severe GA, followed by the group with combined severe WMH and GA. *Post hoc* comparisons (Bonferroni correction) revealed that compared to the group with no or mild WMH and GA, performance was worse in the moderate/severe GA group (*p *< 0.001) and in the group with concurrent moderate/severe WMH and GA (*p *< 0.05). No other between-group comparisons were significant. For executive functioning, performance was lowest in the group with moderate/severe GA only, followed by the group with moderate/severe GA and WMH. *Post hoc* comparisons (Bonferroni correction) revealed that performance was significantly compromised in the moderate/severe GA only group compared to both the no/mild WMH and GA group (*p* < 0.01) and the moderate/severe WMH only group (*p* < 0.05).

Finally, the interaction effect of MTA and GA was significant for episodic memory only (see **Table 3**). Compared to the group with no/mild MTA and GA, episodic memory performance was significantly worse in the moderate/severe MTA only group (*p *< 0.05) and in the moderate/severe GA only group (*p *< 0.01) (Bonferroni correction). No further significant differences were found.

## DISCUSSION

In the present study, we examined the independent effects of WMH and MTA on various cognitive functions in a broad sample of patients seen in a memory clinic, irrespective of diagnosis and without the use of extensive exclusion criteria. Furthermore, we investigated the interaction effect of WMH and MTA on various cognitive functions in this patient sample. The results indicated that MTA was the most important predictor of cognitive function. Main effects of WMH were limited to global cognition, and significant interaction effects were limited to episodic memory. However, in contrast to our expectations, it was not the combination of moderate to severe MTA and WMH that resulted in the worst episodic memory performance, but the presence of moderate to severe MTA without concurrent moderate to severe WMH. This again substantiates the importance of MTA for cognitive test performance. Taken together, the results indicate that MTA is not only associated with episodic memory functioning, as has been found in previous studies, but is also an important correlate of more prefrontally mediated functions, including executive function, psychomotor speed and working memory. Disorders in these functions are often clinically interpreted as evidence of symptomatic cerebrovascular disease, but the current study shows that these functions may be strongly associated with MTA.

### WHITE MATTER DISEASE

Despite extensive research over the last decades, the clinical significance of white matter disease is still a matter of debate. In their review on the relation between WMH and cognitive function, Schmidt et al. (2011) concluded that large inter-individual variability exists with regard to the clinical correlates of WMH. They presumed that one of the reasons for large inter-individual variability in the clinical presentation of subjects with WMH is the complexity of the association. Several factors, such as cognitive reserve and associated loss of brain volume have been identified as mediators between white matter damage and clinical findings. In the current study, support for the clinical relevance of WMH is also limited. Only for global cognition did MTA and WMH contribute independently to poor task performance.

Although the inter-individual variability limits the interpretation of WMH in terms of clinical implications of these MRI findings at an individual level, there is clear evidence that WMH relate to cognitive functioning at a group level. Recent studies show that WMH are associated with deficits in executive functioning, often affecting daily functioning, by impairing erroneous goal formation, planning and organizing (Bracco et al., 2005; Prins et al., 2005; Pohjasvaara et al., 2007; Rabbitt et al., 2007; Tiehuis et al., 2008; Geerlings et al., 2009; Mungas et al., 2009). Beyond executive functioning, WMH seem to have a subtle but noticeable effect on other cognitive domains as well, such as speed of information processing (Prins et al., 2005; Sachdev et al., 2005; van den Heuvel et al., 2006; Delano-Wood et al., 2008; Tiehuis et al., 2008) and memory (Mosley et al., 2005; Au et al., 2006; Chen et al., 2006; Pohjasvaara et al., 2007; Baune et al., 2009; Geerlings et al., 2009). In accordance with these studies we found that WMH were significantly correlated with global cognition, executive function, psychomotor speed and marginally with episodic memory. Nonetheless, when the contributions of MTA and global cortical atrophy were taken into account, the relationship between WMH and cognition remained significant for global cognition only.

### HIPPOCAMPAL ATROPHY

With regard to hippocampal atrophy, MTA measures have shown a relative consistent association with cognitive impairment. For example, our findings are in accordance with previous studies that found that MTA is significantly associated with episodic memory (Kohler, 1994; Laakso et al., 1995; Tulving and Markowitsch, 1998; Stout et al., 1999; Shim et al., 2011). Other cognitive domains have been less exclusively examined in relation to MTA. Only a handful of studies have examined the effect of hippocampal atrophy on executive dysfunction as a non-memory disorder. Recent studies showed a significant relation between hippocampal atrophy and executive dysfunction (Nagata et al., 2010; Oosterman et al., 2012). This finding was confirmed in the present study, in that we found that MTA was also associated with impairments of executive function, as well as global cognitive status (Laakso et al., 1995) and psychomotor speed. In addition, the extent to which the effects of MTA are independent of, and extend beyond, the influence of WMH and global atrophy has been addressed only in few studies.

### DISENTANGLING MTA, GLOBAL ATROPHY, AND WMH CONTRIBUTIONS TO COGNITIVE DYSFUNCTION

Medial temporal atrophy scores are consistently associated with WMH (Appel et al., 2009) and both may be independently associated with cognitive task performance (Stout et al., 1996). However, because MTA has been traditionally linked to memory, and WMH to executive function, the frequent coexistence of MTA and WMH may automatically lead to attribution of all executive dysfunction to WMH and of memory-related dysfunction to MTA. A recent study that examined the independent effects of WMH and MTA on cognition in AD patients (Shim et al., 2011), found that MTA was mostly associated with language and memory, whereas WMH were associated with attention and frontal executive functioning. In this study, however, global atrophy was not taken into account. How this may affect interpretation was shown in a study where WMH were no longer associated with severity of cognitive impairment, after controlling for atrophy (Hirono et al., 2000). The present study confirms this finding: after confounding effects of global atrophy and MTA were controlled for, the unique contribution of WMH to cognitive test performance was minimal. On the other hand, the strong effect of MTA on memory, executive function, psychomotor speed and global cognition, extended beyond potential confounding effects of WMH and global atrophy.

### RELEVANCE

These data are important for our understanding of which diseases or pathological processes underlie cognitive impairment and dementia in the aging population. Although AD is considered the most common cause of dementia, the common view is that dementia in older age is multifactorial, with important contributions of Alzheimer pathology and cerebrovascular disease. Clinically, many such patients are diagnosed as “mixed dementia,” a combination of AD and vascular dementia. This is driven by the high prevalence of WMH on MRI in the aging population. The contribution of these WMH to the observed cognitive dysfunction, however, may be overestimated, as our study indicates. If MTA and global atrophy underlie not only memory, but also executive dysfunction, the clinical impact of WMH may be much smaller than is currently believed. This may also have consequences for preventive strategies. The effect of prevention of WMH may be overestimated if in fact the cognitive impairment attributed to WMH is caused by MTA. For example, optimizing vascular care in AD has been found to be effective in reducing WMH, but had no effect on cognitive function (Richard et al., 2009, 2010).

The present study has some strength and limitations. The strengths of this current study are the large number of subjects evaluated and the detailed neuropsychological, clinical, and MRI investigation in the same subject, gathered within 1 week of examination. Furthermore, the patients enrolled in this study were all first-time referrals by the general practitioner to a local community hospital-based memory clinic. We minimized exclusion criteria to ascertain that the study sample is representative of the population seen in this memory clinic. This is in contrast to the majority of studies that have examined the effects of WMH and MTA on cognitive function that were conducted in strictly defined patients in tertiary or research settings, using extensive exclusion criteria. Our study is the first to show that these findings also apply to a broad sample of patients seen in a general memory clinic. In addition, in contrast to many previous studies, we controlled for global atrophy when examining the unique contributions of WMH and MTA to cognitive task performance.

Despite these strengths, the present study also has some limitations. Although each test was chosen to measure functioning in a specific cognitive domain, there is in fact considerable overlap between cognitive domains. Therefore, reduced performance on a specific test may be caused by impairment in several cognitive domains. For example, the TMT part B is essentially a test for complex psychomotor speed and executive functioning. However, due to the complex test instructions, it also places a substantial demand on memory.

Another potential limitation is that in the current study only a single measure of psychomotor speed was available, which is crucial to take into account since no significant relationship of this cognitive measures with WMH was found. White matter tracts support the functioning of the cognitive processes that reside within different cortical and subcortical brain areas. Consequently, WMH may result in inefficient neural activity that could lead to, in the first instance, cognitive slowing rather than cognitive dysfunction. van den Heuvel et al. (2006) found that the volume of periventricular WMH at baseline was longitudinally associated with more time needed to complete the Stroop test, indicating reduced mental processing speed. Additionally, the progression in periventricular hyperintensity volume paralleled the decline of cognitive slowing. Their set of cognitive tests were somewhat different from the set we used; nevertheless, both sets included a test that has often been used to measure speed of mental processing, the Trail Making Test A. In addition, the strong association between this measure and MTA, together with the fact that the significant correlation between WMH and psychomotor speed disappeared when concurrently considering MTA and GA, indicates that, also for psychomotor speed, the role of WMH may be questionable.

Furthermore, the use of biomarkers to support the presence of AD pathophysiological-process, such as CSF, may be useful as optional clinical tool for diagnostic use. However, at the present time, the access to CSF is limited in our setting for the use of routine diagnostic purposes. Therefore, this variable was not included in our study.

A final possible limitation of the present study is the use of visual rating scales instead of automated volumetric assessment of WMH, MTA, and global atrophy. However, studies have shown high reliability of visual rating scales for assessing WMH and gray matter atrophy (Wahlund et al., 2000; Gouw et al., 2006; Westman et al., 2011). These data indicate that visual rating scales do not underperform compared to volumetric measurements. In our study, a clear benefit of using visual scales is that it reduces exclusion based on MRI imaging quality, as volumetric software is highly sensitive to motion artifacts or small deviations from scanning protocol as often occur in clinical MRI.

To summarize, this study clearly shows that MTA is associated with memory, executive function, psychomotor speed, and global cognitive function, whereas WMH are associated with global cognition; these effects were independent of global cortical atrophy. The relation between morphological abnormalities on MRI and cognitive deficits will continue to be an important topic of neuropsychological and neuroimaging research. The recent development of preventive strategies designed to delay the progression of dementia emphasizes the importance of future research on this subject. These findings set the stage for future studies to clarify the mechanisms that underlie the dementia process.

## Conflict of Interest Statement

The authors declare that the research was conducted in the absence of any commercial or financial relationships that could be construed as a potential conflict of interest.
